# Epithelial CD80 promotes immune surveillance of colonic preneoplastic lesions and its expression is increased by oxidative stress through STAT3 in colon cancer cells

**DOI:** 10.1186/s13046-019-1205-0

**Published:** 2019-05-09

**Authors:** Chiara Marchiori, Melania Scarpa, Andromachi Kotsafti, Susan Morgan, Matteo Fassan, Vincenza Guzzardo, Andrea Porzionato, Imerio Angriman, Cesare Ruffolo, Stefania Sut, Stefano Dall’Acqua, Romeo Bardini, Raffaele De Caro, Carlo Castoro, Marco Scarpa, Ignazio Castagliuolo

**Affiliations:** 1 Department of Molecular Medicine DMM, University of Padua, Padua, Italy; 2 Laboratory of Advanced Translational Research, Veneto Institute of Oncology IOV – IRCCS, via Gattamelata 64, 35128 Padua, Italy; 3 Pathology Unit, Sheffield Teaching Hospitals, Sheffield, UK; 4 Surgical Pathology Unit from the Department of Medicine DIMED, University of Padua, Padua, Italy; 5 Department of Neurosciences, University of Padua, Padua, Italy; 6 General Surgery Unit from the Department of Surgery, Oncology and Gastroenterology DISCOG, University of Padua, Padua, Italy; 7 General Surgery Unit (IV), Ca’ Foncello Hospital, Treviso, Italy; 8 Department of Pharmaceutical and Pharmacological Sciences DSF, University of Padua, Padua, Italy

**Keywords:** Immune surveillance, Colorectal cancer, Dysplasia, CD80

## Abstract

**Background:**

One of the most potent costimulatory molecules involved in the recognition and killing of tumor cells is CD80. However, its role and the molecular mechanisms regulating its expression in sporadic colorectal carcinogenesis remain elusive. Here, we provide evidence for CD80 overexpression in human colon epithelial cells derived from preneoplastic mucosa.

**Methods:**

Expression of CD80 on colonic epithelial cells isolated from normal human colonic mucosa, preneoplastic and neoplastic specimens was assessed by flow cytometry. WT and CD80KO mice received azoxymethane to induce colon preneoplastic lesions and sacrificed to perform histology, flow cytometry analysis and immunohistochemistry of colonic mucosa. Some WT mice were treated with a monoclonal anti-CD80 antibody following AOM administration. Primary colon epithelial cells and CT26 cell line were used to quantify the expression of CD80 in response to pro-oxidant stimuli. Specific pharmacological inhibitors and siRNA silencing were used to inhibit MAPK pathways and STAT3.

**Results:**

CD80 expression was significantly increased in colon epithelial cells of human preneoplastic lesions. In the AOM model, CD80 impairment by administration of neutralizing antibodies or use of CD80 knockout mice enhanced dysplasia development. In vitro, CD80 upregulation was induced by oxidative stress in colon cancer cells and primary colon epithelial cells. In addition, reactive oxygen species could induce CD80 expression via the JNK and p38 MAPK pathways, that activated STAT3 transcription factor in colon cancer epithelial cells.

**Conclusion:**

This study provide evidence for a major role of CD80 in orchestrating immune surveillance of colon preneoplastic lesions and might help to develop novel approaches that exploit anti-tumor immunity to prevent and control colon cancer.

**Electronic supplementary material:**

The online version of this article (10.1186/s13046-019-1205-0) contains supplementary material, which is available to authorized users.

## Background

With more than 1.8 million new cases estimated to occur in 2018, colorectal cancer (CRC) is the third most common cause of cancer-related death worldwide [1]. Despite earlier screenings and improved treatments that significantly dropped the death rates from CRC, there is still need for designing more effective prevention strategies [2].

In the last decade, accumulating evidence supported the concept of immune surveillance as a critical barrier for CRC development, including at the early and premalignant stages, thus it represents an attractive target for early intervention and prevention [3]. Indeed, the infiltration patterns of CD4+, CD8+ TILs, DCs and other immune cells were shown to be progressively altered in the normal-adenoma-carcinoma sequence, and also in the low grades of adenomas [4–7]. Moreover, the presence of CD8+ T cells and increased interferon-gamma (IFNγ) expression were shown to have a better prognostic value than the classic tumor node metastasis classification factor, whereas a T helper 17 (Th17) T-cell-dominated immune response was associated with a worse outcome [8]. Thus, understanding the role and mechanisms of the immune response in colorectal carcinogenesis may provide advances in the development of new immunomodulatory therapeutic strategies and prognostic tools.

One of the most potent costimulatory molecules involved in the recognition and killing of tumor cells is CD80 [9, 10]. It is found not only on dendritic cells, activated B cells, and macrophages [11] but also on non professional antigen presenting cells [12, 13]. Remarkably, CD80 may either activate or inactivate T cells by binding to CD28 or to the cytotoxic T lymphocyte-associated antigen (CTLA-4) receptor, respectively. In vivo evidence for the significance of CD80 in eradication of cancer has been shown by classic tumor immunology studies that have revealed that ectopic expression of CD80 on tumor cells has potent effects on the induction of anti-tumor cytotoxic T lymphocytes (CTL) response [14–16] and sometimes Natural Killer (NK) response [17]. In addition, tumor expression of CD80 also enhances CTL effector functions and facilitates tumor immunity by inhibiting PDL1-mediated immune suppression [18–20]. Although CD80 is not expressed on healthy cells, it can be upregulated within different disease contexts, including infection, transformation, extensive proliferation, wound repair and inflammatory diseases [21]. The molecular pathways directing its inducible expression are still not well defined and depend on transcriptional, translational and post-translational regulation [22, 23].

Our group recently demonstrated that modulation of CD80-CD28 signaling in a murine model of colitis-associated carcinogenesis alters the progression from low grade dysplasia (LGD) to high grade dysplasia (HGD) [12]. Furthermore, we showed that the interaction between CD80+ human colonic epithelial cells and activated CD8+ T cells is required for an effective immune surveillance process in ulcerative colitis associated colon cancer. As sporadic CRC accounts for approximately 70% of CRCs, it should be useful to elucidate the immune mechanisms occurring in the early stages of this process in order to identify new prognostic biomarkers and targets for immunoprevention [24]. Thus, in the present study we aimed at investigating the role of CD80 in colon preneoplastic lesions in vivo and the cellular mechanisms involved in its expression in intestinal epithelial cells in vitro.

## Materials and methods

### Patients

Human mucosa samples comprised 85 patients derived from a prospective series who completed a colonoscopy or underwent colonic resection for either colonic adenoma or CRC. Furthermore, 12 healthy subjects who underwent colonoscopy for colonic cancer screening were recruited as controls. Mucosal samples were obtained from colonic biopsies of normal (sigmoid colon), neoplastic or preneoplastic mucosa (macroscopic lesion). Diagnosis was confirmed by clinical, radiological and histological parameters. The study was performed according to the principles of the Declaration of Helsinki, all participants provided informed consent, and IRB approval (MICCE1 project, Veneto Institute of Oncology, Padova, Italy) was obtained. The characteristics of the patients and controls are outlined in Additional file 1: Table S1.

### Array database meta-analysis

The NCBI-GEO repository of published array data (http://www.ncbi.nlm.nih.gov/geo/) and the GEO2R microarray analysis tool were explored (10th of September 2018) to assess CD80 expression in epithelial cells of sporadic colorectal carcinogenesis cascade (using the keywords: colon AND epithelial cells AND adenoma AND *Homo sapiens*).

### Animal studies

Animal experiments were performed according to Italian Law 26/2014 and European directive 2010/63/UE. Experimental protocols were reviewed and approved by the Institutional Animal Care and Use Committee (“Comitato Etico Scientifico per la Sperimentazione Animale”) of the University of Padova, Padova, Italy. Mice were maintained under standard laboratory conditions with 12:12-h light-dark cycles and free access to regular rodent chow food and water at all stages of the experimental model. C57Bl6/J and B6.129S4-Cd80 tm1Shr/J (CD80 null) mice were purchased from Charles River Laboratories International Inc. (Wilmington, USA). Following previously established methods for inducing colonic neoplasia cohoused 12 weeks old mice received injections of azoxymethane (AOM, Sigma Aldrich, Saint Louis, USA) (10 mg/kg i.p.) once a week for 6 weeks. In a group of C57Bl6/J, anti-CD80 Ab (clone 16-10A1, ATCC hybridoma no. HB-301) was injected i.p. (200 μg/mouse) 12 weeks after the first AOM injection.

All mice were housed in the same animal room and sacrificed 16 weeks after the first AOM injection. Colons were removed, flushed with PBS and the most distal segment above the anus was collected and processed for flow cytometric analysis. Remaining tissue was fixed as “Swiss rolls” in 10% neutral-buffered formalin and paraffin embedded for histology.

### Histopathology

Sections (3 μm) from formalin-fixed and paraffin-embedded mice specimens were stained with hematoxylin-eosin. Histological assessment was performed by a pathologist (S.M.) blinded to the mouse genotype and treatment. The sections were examined for dysplasia and inflammation. Histological inflammation was quantified and classified by a pathologist (S.M.) unaware of the arm of the experiment using Floren’s score and the Vienna classification of gastrointestinal epithelial neoplasia. Murine colons were analysed for dysplasia at high magnification (40x). The extent of dysplasia was quantified as the percentage of involved bowel length.

### Immunohistochemistry

Immunohistochemical analyses were performed using standard procedures. The immunocomplexes were detected using the Real Dako Envision System detection system (Dako, Glostrup, Denmark). The endogenous peroxidase and nonspecific binding were blocked, respectively, with the solution Peroxidase-Blocking Solution and with Protein Block Serum-Free (Dako, Glostrup, Denmark). The Immunohistochemical staining was performed using an antibody against murine CD80 (dilution 1:200; Bioss Antibodies Inc., Woburn, USA) or an anti-phospho-Stat3 (Tyr705) (dilution 1:200, Cell Signaling Technology MA, USA). The reaction was highlighted through the use of the chromogenic substrate 3,3′-diaminobenzidine (DAB) (Dako, Glostrup, Denmark). The sections were counterstained with Mayer’s hematoxylin, subjected to dehydration in increasing solutions of alcohols and xylene, and finally mounted in Dako Mounting Medium.

### Flow cytometry

Human colon mucosa was stripped from the muscularis mucosa, cut into strips, and freed of mucus by a 30-min wash in HBSS containing 10 mM DTT (AppliChem GmbH, Darmstadt, Germany). Colon epithelial cells (CEC) were isolated by 30-min incubation of the mucosa in HBSS containing 1 mM EDTA (Sigma Aldrich). Mice colon samples were minced into 3 to 4 mm pieces with a sterile scalpel and incubated in HBSS supplemented with 1 mM DTT and 0.5 mM EDTA with shaking at 37 °C for 20 min. After washing, tissue pieces were treated with 1 U/ml Dispase (Stemcell Technologies, Vancouver, Canada) in HBSS at 37 °C for 30 min with gentle stirring and then filtered through a sterile stainless steel mesh (pore size 80 μm, Sigma Aldrich) in order to obtain a single-cell suspension. Cell lines treated with H_2_O_2_, pharmacological inhibitors or siRNA were tripsinized and washed with 1X PBS before staining. For staining, 10^5^ cells were suspended in PBS/2% FBS with appropriate combinations of fluorochrome-conjugated antibodies for 30 min on ice. Flow cytometric analysis was performed using a FACSCalibur based on CellQuest software (BD-Becton Dickinson, Franklin Lakes, USA). The antibodies used are summarized in Additional file 2: Table S2.

### Cell culture and treatments

CT26.WT (ATCC CRL-2638TM), HT-29 (ATCC HTB-38) and HCT 116 (ATCC CCL-247) cell lines were grown till 70% confluence in DMEM medium supplemented with 10% FBS, 1% sodium pyruvate and 1% penicillin/streptomycin (all from Gibco-Thermo Fisher Scientific, Waltham, USA) at 37 °C in humidified incubator containing 5% CO_2_. H_2_O_2_ (Sigma Aldrich) working solution was prepared just before adding. Cells were treated with 200 μM H_2_O_2_ for 24 h; when indicated, pharmacological inhibitors (Additional file 3: Table S3) were added to the cell culture 1 h prior to H_2_O_2_ treatment. CT26.WT were transfected with Stat3-C Flag pRc/CMV or pRc/CMV (Invitrogen-Thermo Fisher Scientific) as control. Stat3-C Flag pRc/CMV was a gift from Jim Darnell (Addgene plasmid # 8722). The transfection was performed on 60% confluent cells using Lipofectamine2000 transfection agent (Invitrogen-Thermo Fisher Scientific) according to the manufacturer instructions.

### Isolation and culture of mice colon epithelial cells (CEC)

Following dissection of the colon mucosa into small strips and mucus removal by 1 mM DTT (AppliChem) in HBSS 30 min at room temperature, mucosal strips were incubated in 1 mM EDTA for 10 min at 37 °C. Then, mucosal strips were transferred into fresh culture medium (DMEM with 10% heat inactivated Fetal Bovine Serum (FBS), 2.5% penicillin-streptomycin-Fungizone and 1% gentamicin, all from Gibco-Thermo Fisher Scientific) and after 10 vigorous shakes of the container (this procedure leads to the detachment of IEC in a full-length crypt formation) the IEC crypts solution was transferred to a collagen I-coated (20 μg/cm^2^, Sigma Aldrich) 12-well plate for seeding of the cells. CEC were used 24 h after isolation.

### Detection of apoptosis using the annexin V FITC assay

Apoptosis detection was performed according to manufacturer’s instruction (Annexin V-FITC Apoptosis Detection Kit, eBioscence).

### siRNA transfection

CT26 cell line was transfected with mouse-specific Atm, Atr, Stat3, Stat5a or Stat5b siRNA (OriGene Technologies, Rockville, USA) and non-silencing siRNA (Universal Scrambled Negative Control siRNA Duplex) (OriGene Technologies). The transfection was performed on 60% confluent cells using the RNAimax Lipofectamine transfection agent (Invitrogen-Thermo Fisher Scientific) according to the manufacturer instructions and with 10 nM siRNAs. Transfected cells were incubated in 5% CO_2_ at 37 °C for 24 h; fresh medium was then added along with the addition of 200 μM H_2_O_2_ and the cells were incubated for another 24 h before harvesting for subsequent analysis.

### RNA extraction and qRT-PCR

Total RNA from CT26 and murine colons was isolated using the SV Total RNA Isolation System kit following the manufacturer’s instructions (Promega, Madison, USA). Complementary DNA (cDNA) synthesis was performed using the *iScript*™ cDNA Synthesis Kit (Bio-rad, Hercules CA, USA) according to the manufacturer’s directions. Specific mRNA transcripts were quantified with SYBR Green PCR Master Mix in an ABI PRISM 7000 Sequence Detection System (Applied Biosystems). The expression of the target molecule was normalized to the expression of the 18S housekeeping gene. The specific forward and reverse primers used are for 18S 5′-CTTAGAGGGACAAGTGGCG-3′ 5′-ACGCTGAGCCCAGTCAGTGTA-3′; Cd80 5′-CCCCAGAAGACCCTCCTGATAG-3′ 5′-CGAAGGTAAGGCTGTTGTTTG-3′; Atm 5′-GGAACCAGTTACCATGAATCGTT-3′ 5′-TCTTCAACTTCTTTCACCCTGA-3′; Atr 5′-AGCAAGGTGATCTCATCCGA-3′ 5′-CGACCACCTTTTTCCCATTCG-3′; Stat3 5′-ACTTCAGACCCGCCAACAAA-3′ 5′-CACCACGAAGGCACTCTTCA-3′; Stat5a 5′-CTCCGCAGCACCAGGTAAAC-3′ 5′-GCTGCCCATACAACACTTGC-3′; Stat5b 5′-CTTGTACGGCCAGCATTTCC-3′ 5′-CAAGATCTATTGAGTCCCAGGCT-3′; Nrf2 5′-AGATGACCATGAGTCGCTTGC-3′ 5′-CCTGATGAGGGGCAGTGAAG-3′; Prdx2 5′-GACCTACCTGTGGGACGCTC-3′ 5′-CCACATTGGGCTTGATGGTGT-3′; Prdx6 5′-CTCCAGCTGACAGGCACAAA-3′ 5′-TCGGAGAGGGTGGGAACTAC-3′. Data are presented as a mean fold change over the control.

### Immunofluorescence/measurements of ROS

Cells were washed with PBS 1X and fixed with PFA 4%. Immunofluorescence studies were performed by using antibodies against CD80 (eBioscience Inc.) for 1 h at 37 °C without permeabilization. Then, cells were washed with PBS 1X and slides were mounted and analysed with a confocal laser scanning microscope (Nikon A1R-A1). Image analysis was performed using the Nikon A1R-A1 software. Other immunofluorescence assays were performed by using antibodies against histone H2A.X (Genetex Inc.) and NF-kB p65 (Abcam) for overnight treatment at 4 °C after permeabilization. DRAQ5TM (Thermo Fisher) fluorescent probe solution was used to identify nuclei.

To test the presence of ROS, living cells were stained with 5 μM MitoSOX [3,8-phenanthridinediamine, 5-(6′-triphenylphosphoniumhexyl)-5,6 dihydro-6- phenyl] or 5 μM CM-H2DCFDA (all purchesad from Molecular Probes, Invitrogen, Carlsbad, CA) for 30 mins in CO_2_ incubator at 37 °C, after 30 mins and overnight treatment with pro-oxidants. For all these experiments, slides were mounted and analyzed with a confocal laser scanning microscope (Nikon A1R-A1 or Leica SP2). Image analysis was performed using the Nikon A1R-A1 software or Leica SP2 software.

### Western blotting

CT26 cells were homogenized in RIPA buffer (150 mM NaCl, 50 mM Tris-HCl pH 8.0, 0.1% Triton X-100, 0.1% sodium deoxycholate, 0.1% SDS, 1X protease inhibitors and sodium orthovanadate 1 mM). Particulate material was removed by centrifugation at 4 °C. Protein concentration was determined in each sample using PierceTM BCA protein assay kit (Thermo Fisher Scientific). Twenty μg of total proteins were loaded into the SDS-polyacrylamide gel, along with molecular weight marker. Then transferred onto a nitrocellulose membrane (0.45 μm pore size in roll form, Millipore) and care was taken to remove all air bubbles. The electrophoretic blots were blocked in 5% bovine serum albumin (BSA) or 5% milk in TBST (120 mM Tris-HCl [pH 7.4], 150 mM NaCl, and 0.05% Tween 20) for 1 h at room temperature to saturate additional protein binding sites. Then membranes were incubated overnight a 4 °C with anti phospho-Stat3 (Tyr705) (Cell Signaling Technology, Massachusetts, USA; dilution 1:5000), anti-ATM (Biorbyt Ltd., Cambridge, UK; dilution 1:500), anti-ATR (EMD Millipore, Temecula CA, USA; dilution 1:1000) or anti βactin (Sigma Aldrich, Missouri, USA; dilution 1:500). After membranes washing, they were incubated with anti-rabbit IgG-HRP (Sigma Aldrich, Missouri, USA; dilution 1:5000). Protein bands were visualized using Clarity™ Western ECL substrate substrates (Bio-rad) and images were captured using the Alliance Q9 system (Uvitec, Cambridge, UK). To ensure equal loading and accuracy of changes in protein abundance, protein levels were normalized to beta Actin as housekeeping.

### Statistics

Data are shown as mean +/− SEM. Statistical analysis was performed using GraphPad Prism Software 6.0 (GraphPad Software Inc., La Jolla, USA). Comparisons were performed using Mann–Whitney’s U-test and Student’s t-test in human and in mice/in vitro data, respectively. Differences were considered significant at *p* < 0,05.

Supplementary information is available in Additional file 4: Supplementary Methods.

## Results

### CD80 is overexpressed by epithelial cells in human colon preneoplastic lesions

**Fig. 1:**
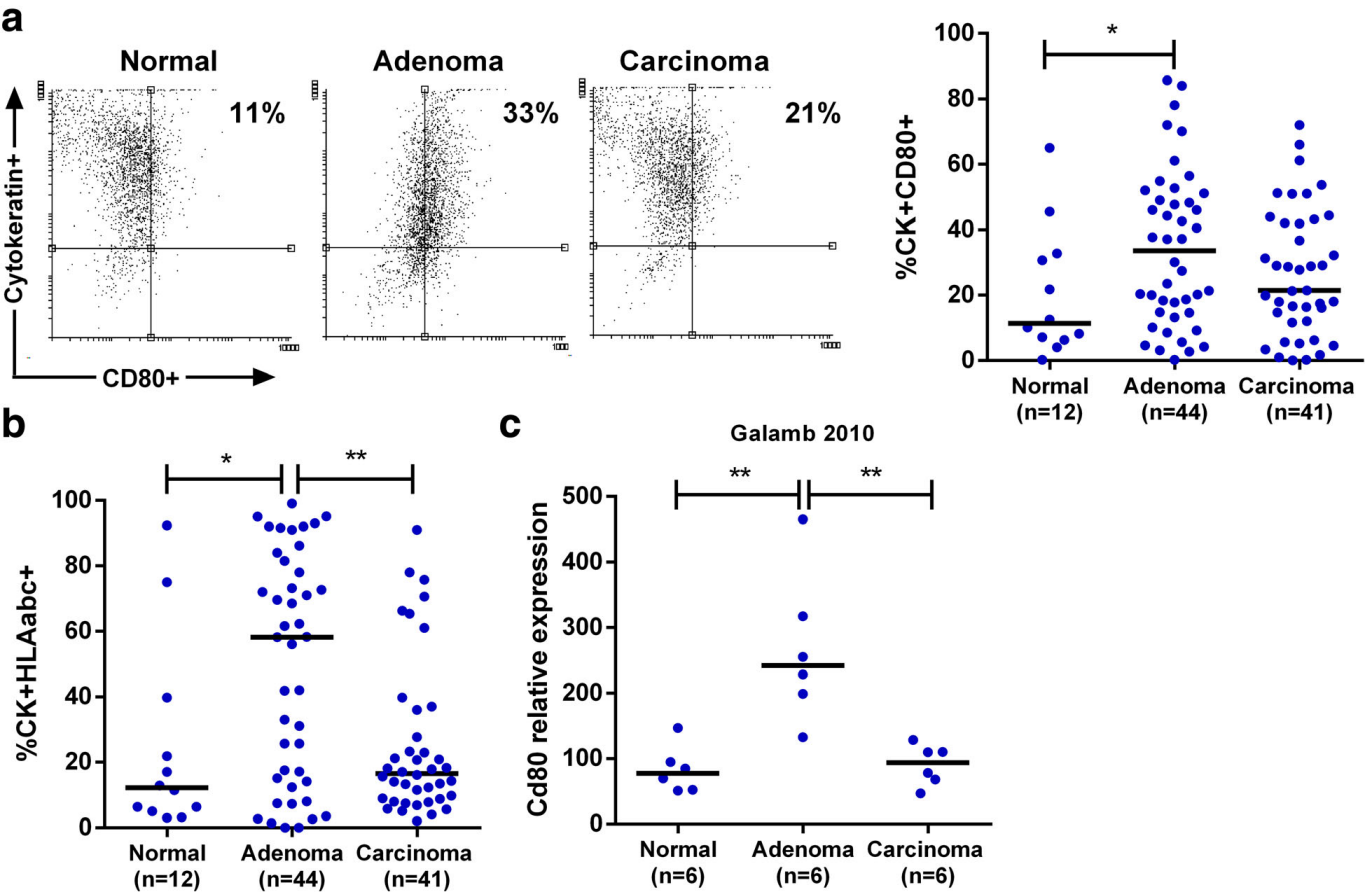
CD80 expression in human colonic epithelial cells. Flow cytometry was performed for surface expression of CD80 (**a**) and HLAabc (**b**) on freshly isolated colon epithelial cytokeratin+ (CK+) cells from human mucosa specimens. Median values are shown. (**c**) An independent study in the NCBI GEO database showed a significant upregulation of CD80 expression (mRNA) in CEC from dysplastic mucosa vs normal or neoplastic mucosa. *P < 0.05 ** *P* < 0.01 by Mann Whitney u test

We have previously shown that CD80 is overexpressed in dysplastic colonic mucosa of UC patients [12]. We wondered whether CD80 would be expressed also in preneoplastic lesions of sporadic colorectal carcinogenesis. To address this question, we compared CD80 expression on colonic epithelial cells (CEC) isolated from normal human colonic mucosa, preneoplastic (i.e. adenoma) and neoplastic (i.e. adenocarcinoma) specimens. We found a significant increase of CD80 expression in epithelial cells of preneoplastic lesions as compared to control tissues (Fig. 1a). Moreover, the percentage of HLA ABC+ CEC was significantly increased in adenoma as compared to control and tumoral mucosa (Fig. 1b). By exploring the NCBI-GEO database, we analysed an independent study on laser microdissected human CEC [25]. The microarray data set (Geo dataset GSE15960) showed a significant up-regulation of CD80 expression in adenoma derived CEC vs normal mucosa derived CEC as well as vs carcinoma derived CEC (Fig. 1c). Overall these data suggest that increased CD80 expression and antigen presenting activity by CEC occurs in precancerous mucosa, prompting further investigation into the role of CD80 in sporadic dysplastic colonic lesions onset.

### CD80 controls the progression of colonic preneoplastic lesions

**Fig. 2:**
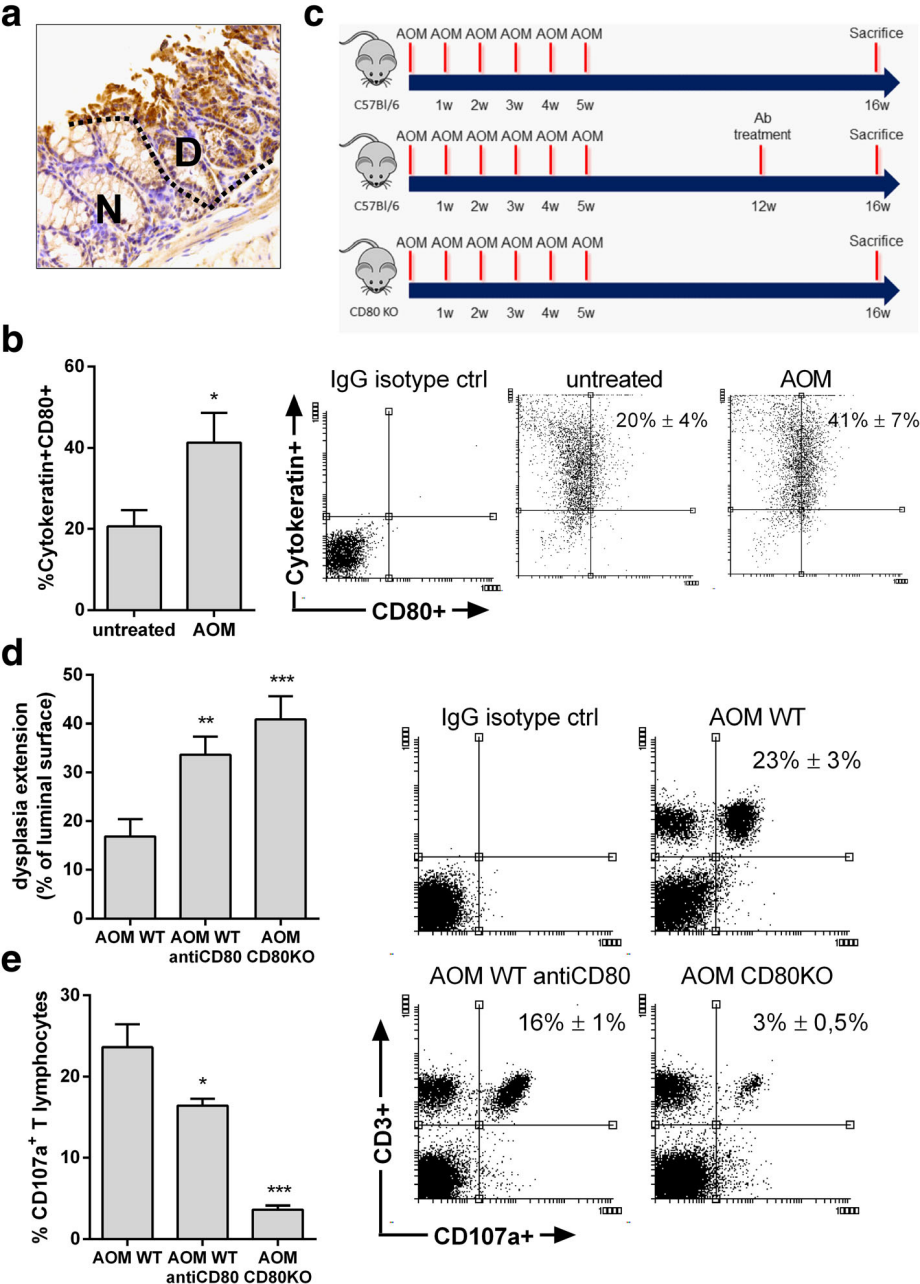
Characterization of CD80 expression in AOM-induced colon dysplasia. Mice were injected with AOM i.p. at a dose of 10 mg/kg body weight and sacrificed 16 weeks after AOM injection (*n* = 15). (**a**) Representative immunohistochemical staining for CD80 of the colon from AOM treated mice exhibiting CD80+ cells in the normal (N) and dysplastic (D) epithelium. (**b**) Representative flow cytometry plots and frequency of CEC isolated from untreated (*n* = 13) and AOM treated mice (n = 15) expressing CD80. (**c**) Scheme for the experimental course of the colon carcinogenesis model in WT and CD80 KO mice and for the administration of neutralizing antibodies in WT mice. (**d**) Extension of dysplasia in AOM-treated WT mice subjected to administration of IgG, anti-CD80 antibodies (200 μg/mouse) and in AOM-treated CD80 KO mice. (*n* = 7–15 mice per group). (**e**) Representative flow cytometry plots and frequency of CD107a + T lymphocytes isolated from colon mucosa of AOM-treated WT mice subjected to administration of IgG, anti-CD80 antibodies (200 μg/mouse) and in AOM-treated CD80 KO mice (n = 7–15 mice per group). Data are presented as mean ± S.E.M. **P < 0.01 *** *P* < 0.001 by unpaired, two-tailed Student’s t-test

Since CD80 expression is increased in the epithelial compartment of preneoplastic lesions, we chose to investigate the functional role of CD80 during the early stages of carcinogenesis in mice. We used the mutagenic agent azoxymethane (AOM), which results in the development of spontaneous preneoplastic lesions within 16 weeks from the first AOM injection. As shown in Fig. 2a, IHC staining showed that epithelial CD80 is differentially expressed by normal vs dysplastic glands of AOM treated mice. Moreover, flow cytometric analysis on CEC showed a significant increase in CD80+ cells frequency in mice treated with AOM vs untreated mice (Fig. 2b). Notably, lack of functional CD80, determined by genetic background in CD80−/− mice or by specific antibody block, caused a significant increase in dysplasia extension in AOM treated mice (Fig. 2d). Moreover, lack of functional CD80 was associated to a significant reduction of CD107a + T cells, a marker of lymphocytes cytotoxic activity (Fig. 2e). Together, these data reveal an active role for CD80 in the prevention of the progression of sporadic colonic early tumorigenesis in vivo.

### Oxidative stress increases CD80 expression in colonic epithelial cells

Our results suggest that CD80 expression is induced in preneoplastic lesions as a protective mechanism against AOM-induced epithelial degeneration. Since AOM causes pathological changes in the colonic mucosa by increasing oxidative stress and consequently genotoxicity [26–28], we hypothesized that CD80 expression is upregulated during colon early carcinogenesis by reactive oxygen species (ROS). Indeed, AOM-treated colonic mucosa was characterized by an oxidative microenvironment, as shown by a significant downregulation of antioxidant genes Nrf2, Prdx2 and Prdx6 and a consistent reduction in the ratio of reduced GSH to oxidized GSH (GSSG), as compared to colonic mucosa of untreated mice (Additional file 5: Figure S1).

**Fig. 3:**
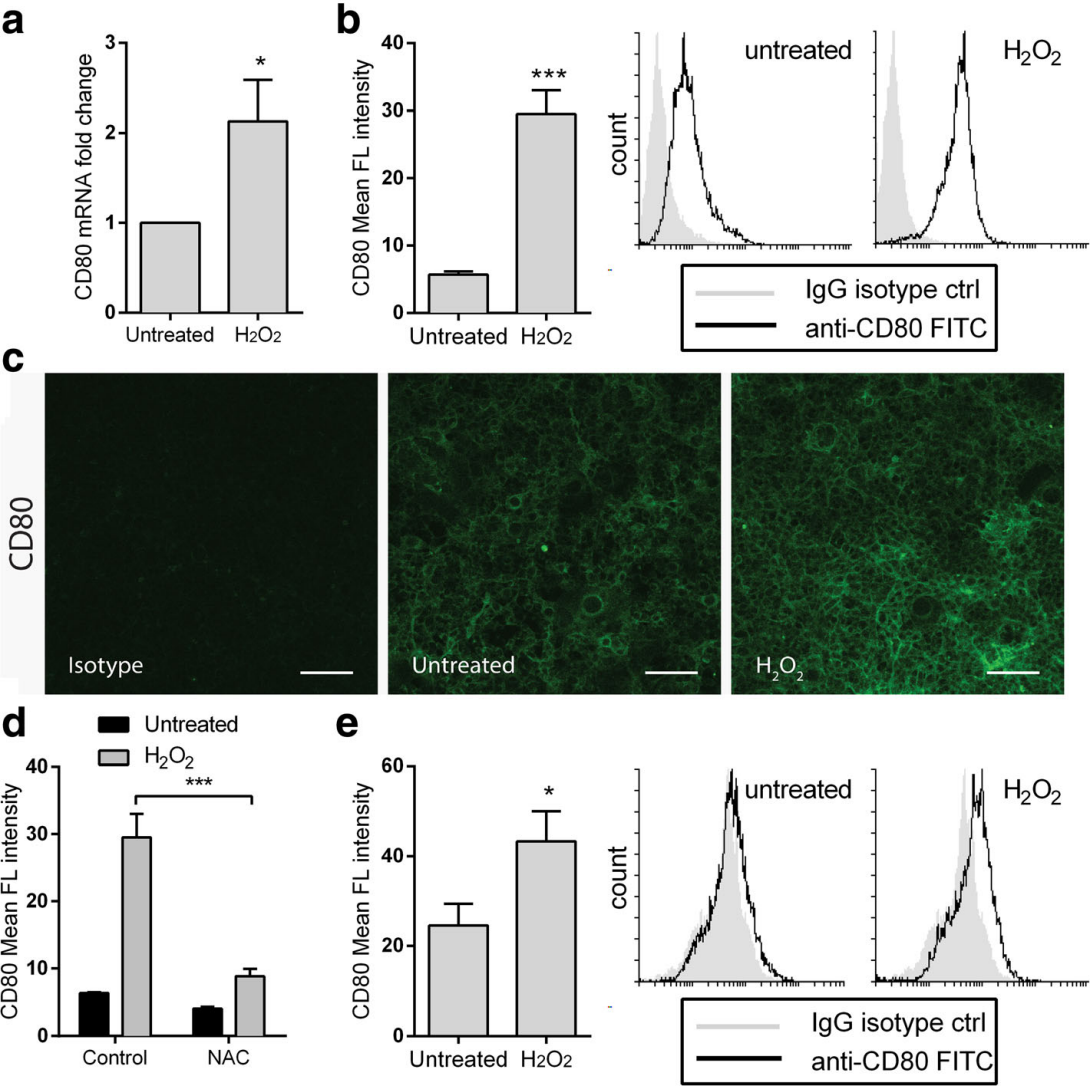
Oxidative stress is a potent inducer of colonic epithelial CD80. (**a**) Real-time PCR for CD80 expression in CT26 cell line after treatment with H_2_O_2_, 200 μM for 6 h. (**b**) Flow cytometry was performed for surface expression of CD80 by CT26 cell line after treatment with H_2_O_2_, 200 μM for 24 h. Representative histogram plots are shown. (**c**) Immunofluorescence staining of CD80 in CT26 cells after 24 h treatment with 200 μM H_2_O_2_. Magnification: 20X. (**d**) Flow cytometry analysis of CT26 cells after 1 h of preincubation with 25 mM N-acetyl cysteine and treatment with H_2_O_2_ (200 μM) for 24 h. (**e**) Primary murine colonic epithelial cells were incubated with H_2_O_2_, 200 μM for 24 h. Flow cytometry was performed for surface expression of CD80. Representative histogram plots are shown. Mean ± S.E.M. of at least three independent experiments is indicated. *P < 0.05 ** *P* < 0.01 by unpaired, two-tailed Student’s t-test

Thus, in order to verify the contribution of ROS to the induction of CD80 expression in CEC, we tested whether H_2_O_2_ could alter the expression of CD80 on CT26 cells. This pro-oxidant agent was able to generate oxidative stress as verified by staining with the fluorogenic dyes MitoSOX and CM-H2DCFDA, which are selective indicators of mitochondrial superoxide and ROS, respectively (Additional file 6: Figure S2). Treatment with H_2_O_2_ significantly increased CD80 expression both at mRNA and protein level (Fig. 3a-c). On the other hand, pre-treatment with N-acetylcysteine (NAC), an antioxidant agent that increases cellular pools of free radical scavengers, prevented H_2_O_2_-mediated CD80 induction, thus confirming a role for free radicals in CD80 upregulation (Fig. 3d). Moreover, CD80 expression was upregulated also in primary murine CEC following treatment with ROS-generating agent as detected by flow cytometry analysis (Fig. 3e), supporting oxidative stress as a key regulator of CD80 expression in CEC.

### CD80 induction by oxidative stress is not a consequence of apoptosis or NF-kB signalling in colon cancer cells

**Fig. 4:**
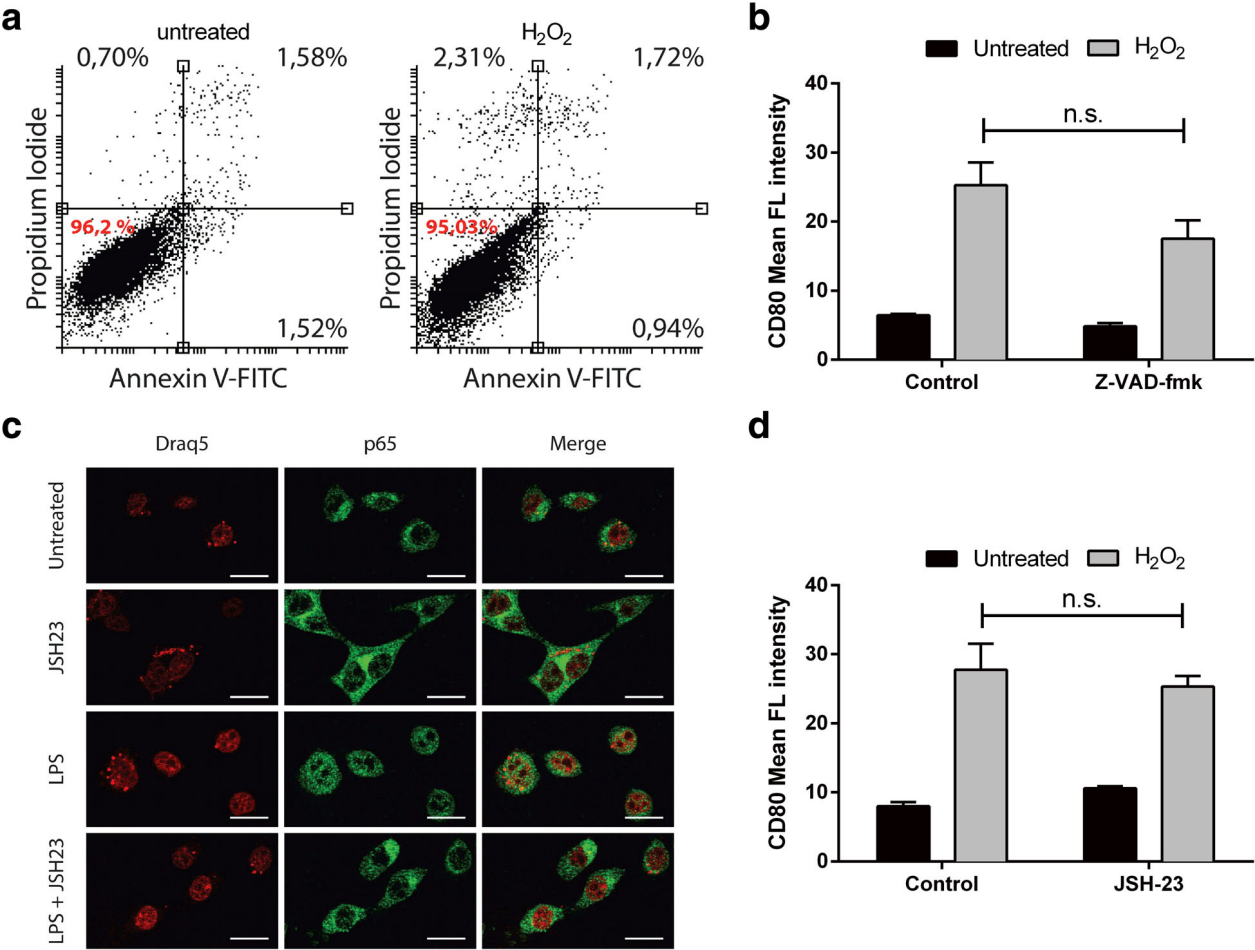
Apoptosis and NF-ĸB signalling are not involved in oxidative stress-mediated CD80 induction. (**a**) CT26 cells were incubated with H_2_O_2_ 200 μM for 24 h and assayed for annexin V by flow cytometry. (**b**) Flow cytometry analysis of CT26 cells after 1 h of preincubation with 25 μM Z-VAD-fmk and treatment with H_2_O_2_ (200 μM) for 24 h. (**c**) Immunofluorescence staining of p65 in CT26 cells after 24 h treatment with 10 μM JSH-23, 1 μg/ml LPS or JSH-23 + LPS together. Magnification: × 60. (**d**) Flow cytometry analysis of CT26 cells after 1 h of preincubation with 10 μM JSH-23 and treatment with H_2_O2 (200 μM) for 24 h. Mean ± S.E.M. of at least three independent experiments is indicated. *P < 0.05 ** P < 0.01 by unpaired, two-tailed Student’s t-test

Next, we explored which pathways are involved in oxidative stress mediated CD80 upregulation in CEC. Previous studies showed that following exposure to H_2_O_2_, CEC are primed for cell death. Thus, it was possible that the induction of CD80 was a result of oxidative-stress induced apoptosis. In our experiment, 24 h treatment with 200 μM H_2_O_2_ did not cause a significant increase in Annexin V+ CT26 cells (Fig. 4a). Moreover, treatment with Z-VAD-fmk. a pan-caspase inhibitor, did not prevent H_2_O_2_-mediated CD80 induction, thus ruling out the possibility that CD80 induction is a consequence of apoptosis (Fig. 4b). Nuclear factor-kappaB (NF-κB) signalling is another of the key regulatory pathways classically activated by oxidative stress that could be involved in CD80 induction. We pharmacologically blocked NF-ĸB nuclear translocation using the inhibitor JSH-23 in H_2_O_2_-treated CT26 cells. As expected, JSH-23 inhibited LPS-induced nuclear translocation of the p65 subunit of NF-ĸB (Fig. 4c). However, H_2_O_2_-induced CD80 up-regulation in CEC was not affected, suggesting that CD80 induction is not NF-ĸB signalling dependent (Fig. 4d).

### DNA damage response is not required for the induction of CD80 by ROS in colon cancer cells

**Fig. 5:**
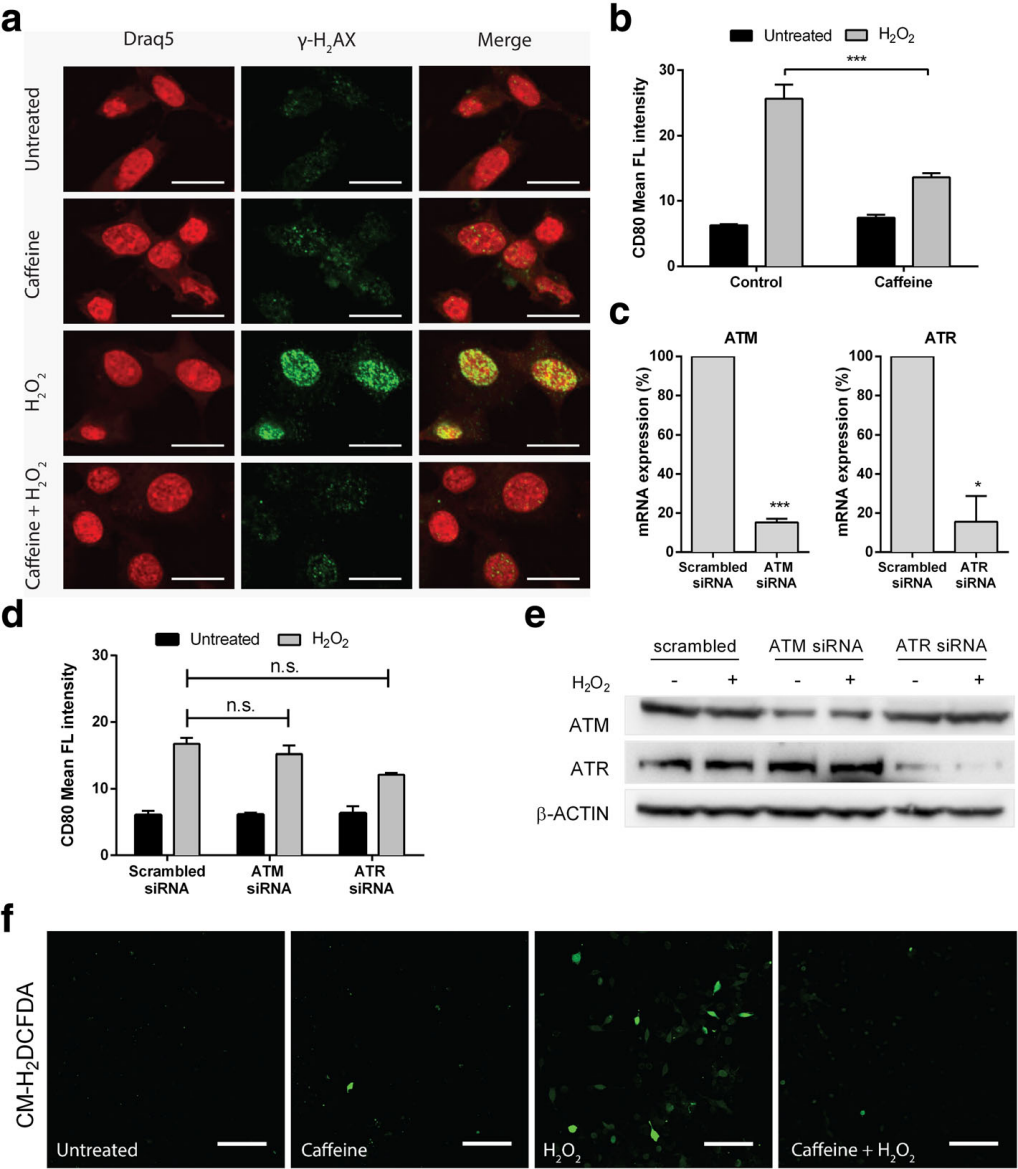
CD80 induction by oxidative stress is not a consequence of DNA damage response. (**a**) Immunofluorescence staining of γH2AX in CT26 cells after 24 h treatment with 5 mM Caffeine, 200 μM H_2_O_2_ or Caffeine+H_2_O_2_ together. Magnification: × 40. (**b**) Flow cytometry analysis of CT26 cells after 1 h of preincubation with 5 mM Caffeine and treatment with H_2_O_2_ (200 μM) for 24 h. (**c**) CT26 cells were transfected with control, ATM and ATR siRNAs. After 24 h, silencing efficiency was tested by RT Real Time PCR. (**d**) CT26 cells were transfected with control, ATM and ATR siRNAs. After 24 h, cells were treated with 200 μM H_2_O_2_ for 24 h before flow cytometry for CD80. (**e**) CT26 cells were transfected with control, ATM or ATR siRNAs. After 24 h, cells were treated with 200 μM H_2_O_2_ for 24 h before western blotting for ATM or ATR. (**f**) Representative staining of CT26 cells with the fluorogenic dye CM-H2DCFDA after 1 h of preincubation with 5 mM Caffeine and treatment with H_2_O_2_ (200 μM) for 1 h. Magnification: 40X. Mean ± S.E.M. of at least three independent experiments is indicated. n.s. not significant, *P < 0.05 ** P < 0.01 *** P < 0.001 by unpaired, two-tailed Student’s t-test

Because H_2_O_2_ is known to provoke DNA damage, it was possible that the H_2_O_2_ -mediated CD80 up-regulation was a consequence of the activation of the DDR (DNA damage response). Indeed, the induction of ɣH2AX, a reliable marker of DDR, occurred after H_2_O_2_ treatment in CT26 cell line (Fig. 5a). This induction was completely blocked by caffeine, a known inhibitor of the ATM/ATR, the two kinases activated by DDR. Remarkably, flow cytometry results indicated that caffeine effectively decreased H_2_O_2_-induced CD80 expression in CT26 cells, too (Fig. 5b). However, selective depletion of ATM or ATR by siRNA failed to block H_2_O_2_ mediated CD80 induction (Fig. 5c-e).

Taken together, our data show that CD80 overexpression induced by ROS is not DNA damage response dependent and the results obtained with the use of caffeine were probably due to its capacity in decreasing generation of reactive oxygen species (Fig. 5f; [29, 30]).

### Oxidative stress mediated CD80 induction relies on STAT3 transcription factor through MAPK activation in colon cancer cells

**Fig. 6:**
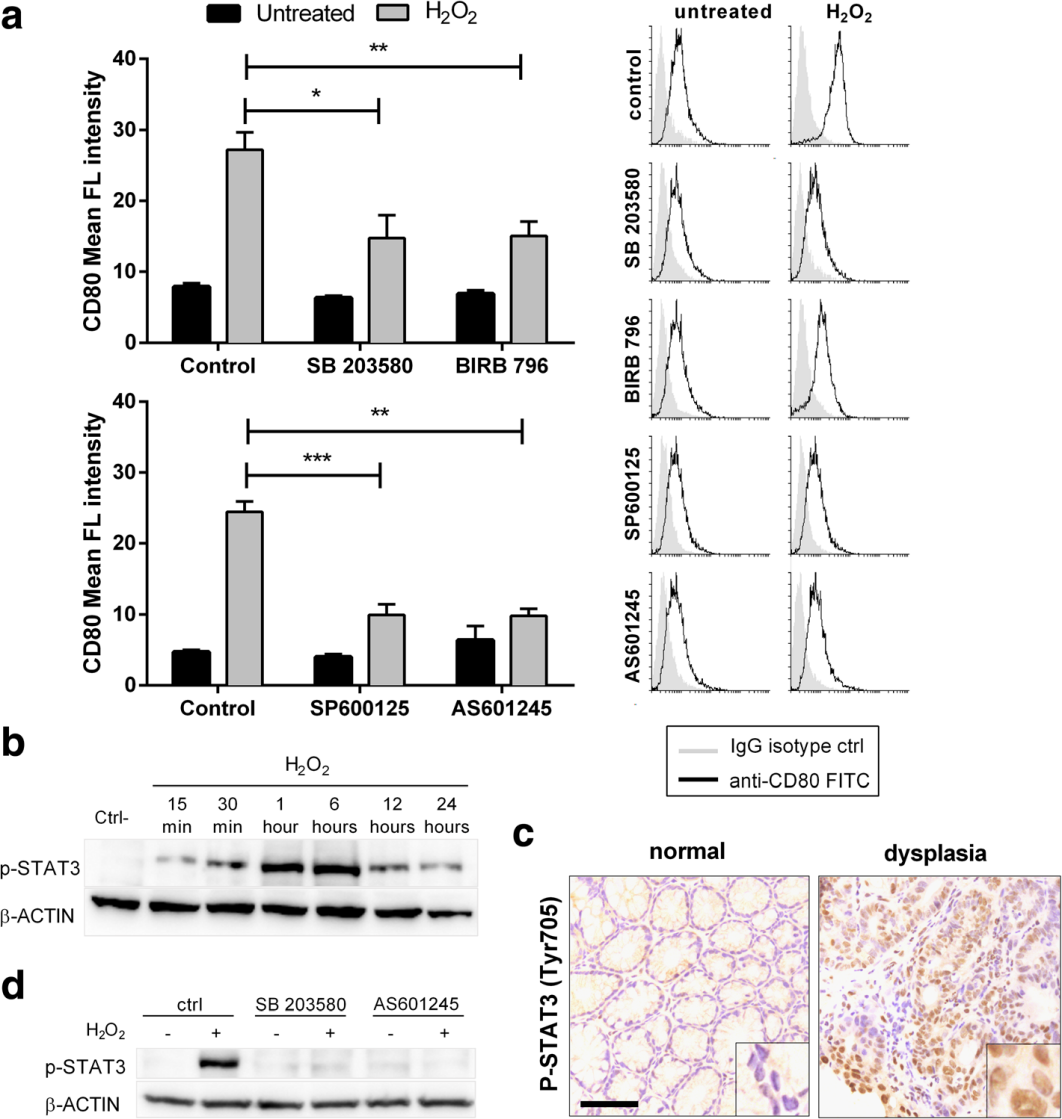
CD80 upregulation is mediated by MAPK activation. (**a**) Flow cytometry analysis of CT26 cells after 1 h of preincubation with p38 inhibitors (5 μM SB203580 and 5 μM BIRB796) or with JNK inhibitors (10 μM SP600125 and 1 μM AS601245) and treatment with H_2_O_2_ (200 μM) for 24 h. Representative histogram plots are shown. (**b**) Western blotting for phosphoSTAT3 of CT26 cells after treatment with 200 μM H_2_O_2_. (**c**) Representative immunohistochemical staining for phosphoSTAT3 of the colon from AOM treated mice exhibiting activated STAT3 cells in the dysplastic epithelium (100 μm bar). (**d**) Western blotting for phosphoSTAT3 of CT26 cells after 1 h of preincubation with p38 inhibitor (5 μM SB203580) or with JNK inhibitor (1 μM AS601245) and treatment with H_2_O_2_ (200 μM) for 1 h. Mean ± S.E.M. of at least three independent experiments is indicated. *P < 0.05 ** P < 0.01 by unpaired, two-tailed Student’s t-test

**Fig. 7:**
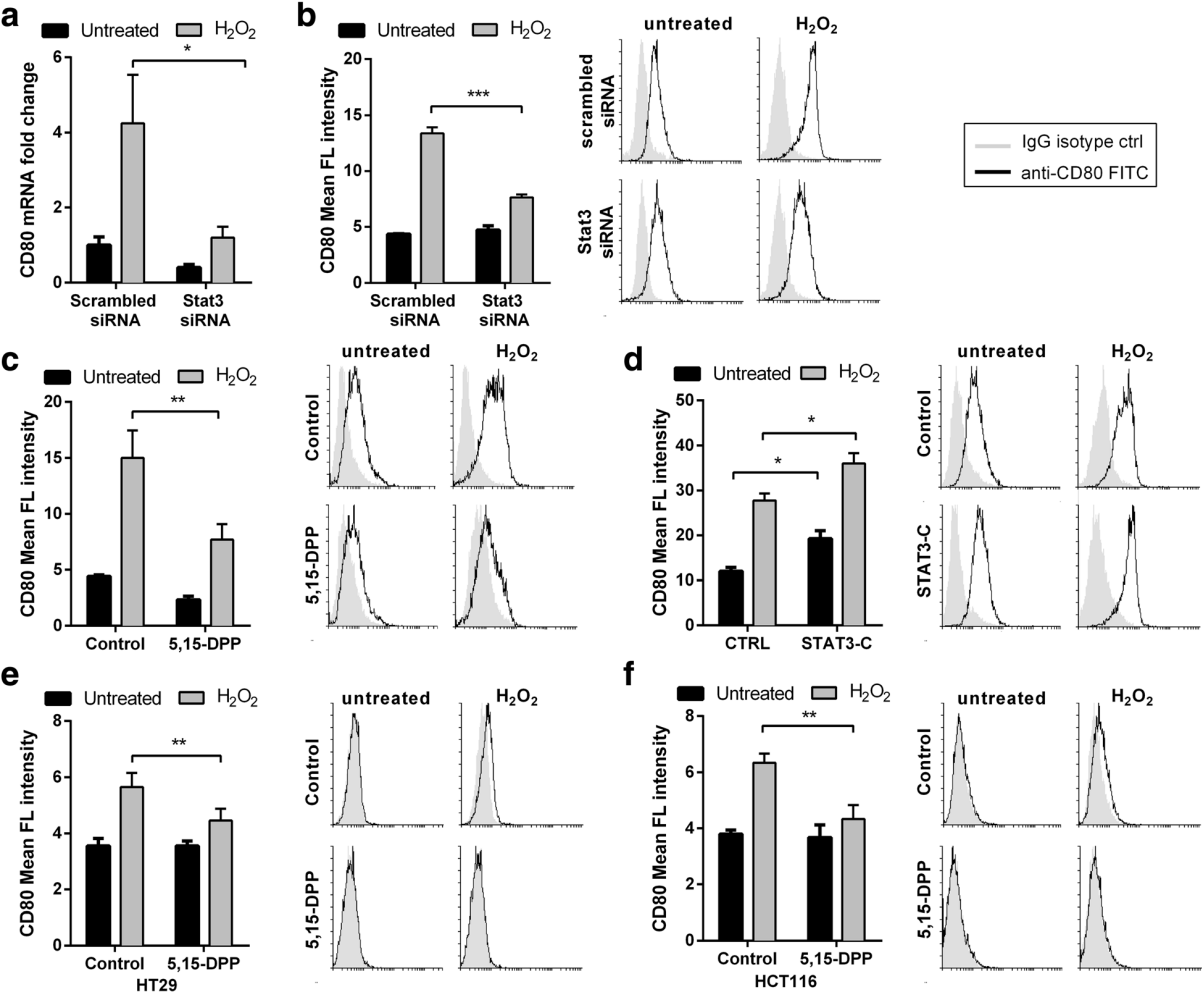
CD80 upregulation is mediated by STAT3 activation. CT26 cells were transfected with control or STAT3 siRNAs. After 24 h, cells were treated with 200 μM H_2_O_2_ for 24 h before qReal Time PCR (**a**) or flow cytometry (**b**) for CD80. (**c**) Flow cytometry analysis of CT26 cells after 1 h of preincubation with 5 μM 5,15-DPP and treatment with H_2_O_2_ (200 μM) for 24 h. (**d**) CT26 cells were transfected with plasmid vector alone (CTRL) or to overexpress constitutively active STAT3 (STAT3C); 24 h after transfection, cells were treated with 200 μM H_2_O_2_ for additional 24 h before flow cytometry for CD80. Flow cytometry analysis of (**e**) HT-29 and (**f**) HCT 116 cells after 1 h of preincubation with 5 μM 5,15-DPP and treatment with H2O2 (200 μM) for 24 h. Representative histogram plots are shown for all flow cytometry experiments. Mean ± S.E.M. of at least three independent experiments is indicated. *P < 0.05 ** P < 0.01 by unpaired, two-tailed Student’s t-test

Two of the major mitogen-activated protein kinase (MAPK) pathways, c-Jun-N-terminal kinases (JNKs) and p38, are known to be activated by oxidative stress. Thus, we suppressed the kinases activity by using two selective pharmacologic MAPKs inhibitors with unrelated chemical structure for each kinase (SB203580 and BIRB796 for p38, SP600125 and AS601245 for JNK). All the inhibitors partially prevented H_2_O_2_ induced CD80 up regulation, demonstrating that both p38 and JNK are involved in CD80 induction (Fig. 6a). Since the activation of the transcription factor STAT3 can occur via phosphorylation by MAPK, we checked its status in CT26 upon H_2_O_2_ treatment. We observed that oxidative stress activated STAT3, as shown by increased levels of phosphorylation on Tyr705 (Fig. 6b). Notably, STAT3 activation was also observed in vivo in the dysplastic epithelial glands of AOM treated mice, as shown by IHC staining (Fig. 6c). Thus, we tested MAPKs inhibitor effect on it. Indeed, oxidative stress mediated STAT3 activation was abolished by CEC treatment with both p38 and JNK inhibitors (Fig. 6d). Next, we started investigating the role of STAT3 on CD80 expression. STAT3 knockdown by siRNA in CT26 cell line significantly decreased CD80 expression induced by free radicals at both mRNA and protein level (Fig. 7a and b). The pharmacological inhibition of STAT3 using 5,15-DPP prevented oxidative stress mediated CD80 induction, too (Fig. 7c). Furthermore, CT26 cells overexpressing constitutively active STAT3 expressed augmented basal levels of CD80 and addition of H_2_O_2_ enhanced CD80 expression compared to control cells (Fig. 7d). Finally, we verified whether oxidative stress mediated CD80 induction relied on STAT3 also in human colon epithelial cell lines. As shown in Fig. 7e and f, pharmacological inhibition of STAT3 prevented the increase of CD80 expression after ROS addition in both HT-29 and HCT 116 cells, respectively. Since STAT3 inhibition did not completely abolished oxidative stress mediated CD80 induction in CT26, we tested also other members of the STAT family, STAT5a and STAT5b, which can be activated by oxidative stress and have been involved in CD80 expression regulation [31]. Notably, the knockdown of both transcription factors did not affect CD80 expression upon ROS stimulation (Additional file 7: Figure S3). Altogether, these data suggest that ROS induce CD80 expression via MAPK pathways that activate STAT3 in colon cancer epithelial cells.

## Discussion

Immunosurveillance represents a critical barrier that emerging tumor cells have to overcome in order to sustain the course of tumor development. Considering the immune-modulating effects of chemopreventive agents as well as the recent success of cancer immunotherapy, the identification of molecules that regulate immunosurveillance mechanism during the early stages of carcinogenesis is pivotal to develop immunotherapeutic approaches and new prognostic biomarkers. Here we provide evidence for a critical role of the costimulatory molecule CD80 in the progression of sporadic colorectal carcinogenesis. Our finding that human CEC from preneoplastic lesions overexpress CD80 and HLA ABC molecules compared to normal and tumor-derived CEC fosters the hypothesis that dysplastic cells can be recognized and eliminated in human sporadic CRC. Indeed, a large fraction of the genomic, epigenomic, and proteomic alterations in CRCs are acquired at early stages [32–34]. The distinct types and considerable extent of these alterations suggest they would necessarily be detected by the immune system. However, very little is known about how these alterations lead to the full activation of the immune response. Our experimental model allowed us to demonstrate that interfering with early CD80 signaling - by neutralizing antibodies administration or use of a knockout strain - results in a significant augmentation of dysplasia extension in the colonic mucosa of AOM-treated mice. This is likely due to the impairment of the induction of a CTL and NK response against emerging dysplastic epithelial cells [14–19].

Little is known about CD80 expression regulation by non–bone marrow–derived cells. In human keratinocytes, CD80 gene expression is upregulated by allergens and irritants [35]. In the kidney, CD80 can be induced in glomerular endothelial cells by warm ischemia/reperfusion injury in rats [36]. Furthermore, CD80 up-regulation has been detected in mouse tumors cell lines treated with radiotherapy [37]. In general, these reports suggest the inducibility of CD80 under stress conditions. This is in line with the results we obtained in our in vitro experiments, that showed a strong and robust induction of CD80 in human [38] and murine colon epithelial cells upon oxidative stress. Indeed, during colonic carcinogenesis, reactive oxidants can be generated from both endogenous and exogenous sources such as infiltrating inflammatory cells, an oncogenic insult and from commensal bacterial metabolism. Notably, it has been shown that oncogenic WNT activation triggers ROS production in CEC [39] and that gut flora may modulate epithelial cells function through extracellular free radical production [40, 41]. On the other hand, downregulation/loss of CD80 in cancer cells seem to occur mainly through epigenetic mechanisms. A study reported hypermethylation of the CD80 promoter in mice tumors [23] and we showed that CD80 down-regulation is associated to aberrant DNA methylation in non-inflammatory colon carcinogenesis [42]. Furthermore, the miR-132-3p, miR-212-3p, and miR-361-5p binding sites in the CD80 gene have been involved in the development and progression of gastric cancer [22] whereas miR-424(322) affects the immune regulation and drug resistance of ovarian cancer by targeting CD80 and CD274 (PD-L1) [43].

Our data demonstrated that in colon cancer cells CD80 induction by oxidative stress was mediated by two different MAPK pathways converging to the transcription factor STAT3. The role of STAT3 in carcinogenesis is controversial: studies have demonstrated that STAT3 can function either as an oncoprotein or a tumor suppressor in the same cell type, depending on the specific genetic background or presence/absence of specific coexisting biochemical defects [44]. In the AOM and the Apc (Min/+) mouse models of colorectal cancer, the deletion of STAT3 in the intestinal epithelial cells reduced early adenoma formation (i.e., oncogenic role) [45, 46]. However, ablation of STAT3 in the later stage of tumor progression significantly increased the invasiveness of the tumors and decreased the survival of the animals (i.e., tumor suppressor role) [45]. Since low surface expression of CD80 is an immunoescape mechanism of colon carcinoma [47], we speculate that the tumor suppressor role of STAT3 in the later stages of colon cancer progression may be explained by its ability to enhance CD80 expression.

## Conclusions

Our findings shed new light onto the complex regulation in colon epithelial cells of the costimulatory molecule CD80, that proved to be an important mediator of immune defense against colon cancer development, and might provide the basis for novel strategies that exploit anti-tumor immunity to prevent and/or control colon cancer.

## Additional files

Additional file 1: **Table S1.** Patients’ characteristics. (DOCX 15 kb)

Additional file 2: **Table S2.** Antibodies used for flow cytometry. (DOCX 12 kb)

Additional file 3: **Table S3.** Pharmacological inhibitors used in the study. (DOCX 12 kb)

Additional file 4: **Supplementary Methods.** LC-MS/MS analysis of GSH-GSSG. (DOCX 13 kb)

Additional file 5: **Figure S1.** Oxidative microenvironment in the colonic mucosa of AOM-treated mice. (**a**) Real-time PCR for Nrf2, Prdx2 and Prdx6 expression in the colonic mucosa of mice treated with AOM (*n* = 6) and untreated mice (*n* = 5). (**b**) Reduced and oxidized GSH were measured by HPLC in the colonic mucosa of mice treated with AOM (*n* = 4) and untreated mice (*n* = 7). Data are presented as mean ± S.E.M. **P < 0.01 *** *P* < 0.001 by unpaired, two-tailed Student’s t-test. (TIF 125 kb)

Additional file 6: **Figure S2.** Oxidative stress in CT26 cells. Representative staining of CT26 cells with the fluorogenic dyes MitoSOX and CM-H2DCFDA after 30 min and O/N treatment with 200 μM H_2_O_2_. Magnification: 40X. (TIF 7723 kb)

Additional file 7: **Figure S3.** CD80 induction by oxidative stress is not mediated by STAT5. (**a**) CT26 cells were transfected with control, STAT5a or STAT5b siRNAs. After 24 h, silencing efficiency was tested by RT Real Time PCR. (**b**) CT26 cells were transfected with control, STAT5a or STAT5b siRNAs. After 24 h, cells were treated with 200 μM H2O2 for 24 h before flow cytometry for CD80. Data are presented as mean ± S.E.M. **P < 0.01 *** P < 0.001 by unpaired, two-tailed Student’s t-test. (TIF 280 kb)

## Abbreviations

AOM: Azoxymethane
CEC: Colon epithelial cells
CRC: Colorectal cancer
CTL: Cytotoxic T lymphocytes
HGD: High grade dysplasia
LGD: Low grade dysplasia
NK: Natural killer
ROS: Reactive oxygen species
