# Supplementary material for: Epithelial CD80 promotes immune surveillance of colonic preneoplastic lesions and its expression is increased by oxidative stress through STAT3 in colon cancer cells
Source: J Exp Clin Cancer Res. 2019 May 9;38:190. doi: 10.1186/s13046-019-1205-0 (PMC6509793; doi:10.1186/s13046-019-1205-0)
Supplement: Supplementary file 1 — Table S1. Patients’ characteristics. (DOCX 15 kb) [file 13046_2019_1205_MOESM1_ESM.docx]

**Table S1. Patients’ characteristics**

|  | Control subjects  (N=12) | Adenoma  (N=44) | CRC  (N=41) |
| --- | --- | --- | --- |
| Gender (F/M) | 4/8 | 15/29 | 20/21 |
| Median Age (IQR) | 59 (50-66) | 65 (55,5-75) | 68 (62,75-75,75) |
