# Supplementary material for: Epithelial CD80 promotes immune surveillance of colonic preneoplastic lesions and its expression is increased by oxidative stress through STAT3 in colon cancer cells
Source: J Exp Clin Cancer Res. 2019 May 9;38:190. doi: 10.1186/s13046-019-1205-0 (PMC6509793; doi:10.1186/s13046-019-1205-0)
Supplement: Supplementary file 2 — Table S2. Antibodies used for flow cytometry. (DOCX 12 kb) [file 13046_2019_1205_MOESM2_ESM.docx]

**Table S2**. Antibodies used for flow cytometry.

| **Antibody** | **Source** | **Clone** |
| --- | --- | --- |
| Anti-mouse CD80 FITC | eBioscience Inc., San Diego, CA | 16-10A1 |
| Anti-human CD80 FITC | eBioscience Inc., San Diego, CA | 2D-10 |
| Anti-human HLA ABC FITC | eBioscience Inc., San Diego, CA | W6/32 |
| Anti-human/mouse pan Cytokeratin PE | Abcam Ltd., Cambridge, UK | C-11 |
| Anti-mouse CD3 APC | eBioscience Inc., San Diego, CA | 17A2 |
| Anti-mouse CD107a PE | eBioscience Inc., San Diego, CA | eBio1D4B |
