# Supplementary material for: Epithelial CD80 promotes immune surveillance of colonic preneoplastic lesions and its expression is increased by oxidative stress through STAT3 in colon cancer cells
Source: J Exp Clin Cancer Res. 2019 May 9;38:190. doi: 10.1186/s13046-019-1205-0 (PMC6509793; doi:10.1186/s13046-019-1205-0)
Supplement: Supplementary file 3 — Table S3. Pharmacological inhibitors used in the study. (DOCX 12 kb) [file 13046_2019_1205_MOESM3_ESM.docx]

**Table S3.** Pharmacological inhibitors used in the study.

| **Chemicals** | **Manufacturer** | **Final Concentration** |
| --- | --- | --- |
| N-acetyl cysteine | Sigma-Aldrich (St. Louis, MO, USA) | 25 mM |
| Z-vad-fmk | Cayman Chemical (Ann Arbor, MI, USA) | 25 μM |
| Caffeine | BioVision Inc. (San Francisco, CA, USA) | 5 mM |
| AS601245 | Merck Millipore (Billerica, MA, USA) | 1 μM |
| SP600125 | Sigma-Aldrich (St. Louis, MO, USA) | 10 μM |
| JSH-23 | Sigma-Aldrich (St. Louis, MO, USA) | 10 μM |
| SB203580 | Sigma-Aldrich (St. Louis, MO, USA) | 5 μM |
| 5, 15-DPP | Sigma-Aldrich (St. Louis, MO, USA) | 5 μM |
| BIRB 796 | Merck Millipore (Billerica, MA, USA) | 5 μM |
