# Supplementary material for: Epithelial CD80 promotes immune surveillance of colonic preneoplastic lesions and its expression is increased by oxidative stress through STAT3 in colon cancer cells
Source: J Exp Clin Cancer Res. 2019 May 9;38:190. doi: 10.1186/s13046-019-1205-0 (PMC6509793; doi:10.1186/s13046-019-1205-0)
Supplement: Supplementary file 4 — Supplementary Methods. LC-MS/MS analysis of GSH-GSSG. (DOCX 13 kb) [file 13046_2019_1205_MOESM4_ESM.docx]

**SUPPLEMENTARY MATERIALS AND METHODS**

**LC-MS/MS analysis of GSH-GSSG**

For LC-MS/MS analysis, and Agilent 1260 chromatograph equipped with MS320 triple quadrupole mass spectrometer using a published protocol with minor modifications (1). Electro Spray ionization (ESI) source was used and measure was operated in multiple reactions monitoring (MRM) in positive ion mode. The MRM for GSH (m/z 308.1→m/z 76.2 + 84.2 + 161.9) and GSSG (m/z 613.2→m/z 230.5 + 234.6 + 354.8) were obtained using optimized collision energy. Limit of Detection (LOD) and Limit of Quantification (LOQ) were 0,3 ng/mL and 1.1 ng/mL respectively. Exactly weighted (±0.1 mg) samples aliquots of 10-50 mg of mice colonic mucosa were extracted with 100μL of a mixture of trichloroacetic acid 10% with 1mM EDTA in Falc ultrasound bath for 10 minutes at room temperature. The samples were centrifuged at 13000 rpm for 15 minutes and supernatant used for analysis. A Phenomenex Kinetek F5 (50 x 3 mm) 2,6 micron column was used. The mobile phases were 1% formic acid in water and methanol. Separation was performed under gradient conditions from 95% of water to 95% methanol.

1. Squellerio I, Caruso D, Porro B, Veglia F, Tremoli E, Cavalca V. Journal of Pharmaceutical and Biomedical Analysis Direct glutathione quantification in human blood by LC – MS / MS : comparison with HPLC with electrochemical detection. *J Pharm Biomed Anal*. 2012;71:111-118. doi:10.1016/j.jpba.2012.08.013
