# Supplementary figures and images for: Epithelial CD80 promotes immune surveillance of colonic preneoplastic lesions and its expression is increased by oxidative stress through STAT3 in colon cancer cells
Source: J Exp Clin Cancer Res. 2019 May 9;38:190. doi: 10.1186/s13046-019-1205-0 (PMC6509793; doi:10.1186/s13046-019-1205-0)

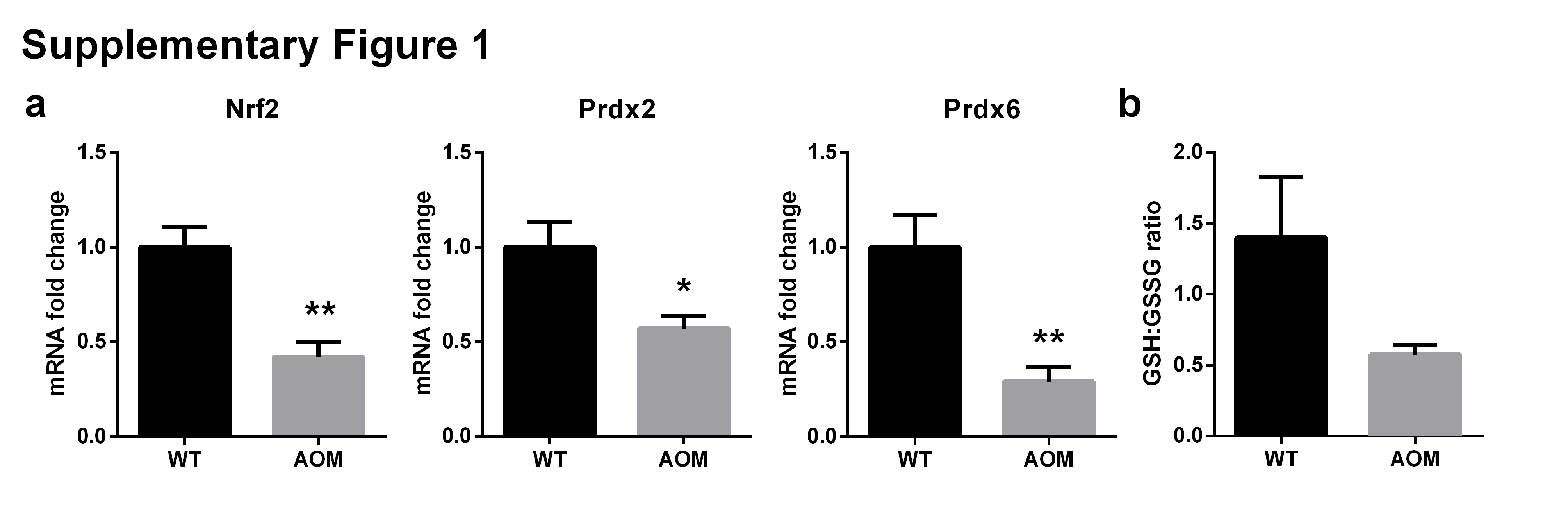

Supplement: Supplementary file 5 — Figure S1. Oxidative microenvironment in the colonic mucosa of AOM-treated mice. (a) Real-time PCR for Nrf2, Prdx2 and Prdx6 expression in the colonic mucosa of mice treated with AOM (n = 6) and untreated mice (n = 5). (b) Reduced and oxidized GSH were measured by HPLC in the colonic mucosa of mice treated with AOM (n = 4) and untreated mice (n = 7). Data are presented as mean ± S.E.M. **P < 0.01 *** P < 0.001 by unpaired, two-tailed Student’s t-test. (TIF 125 kb) [file 13046_2019_1205_MOESM5_ESM.tif]

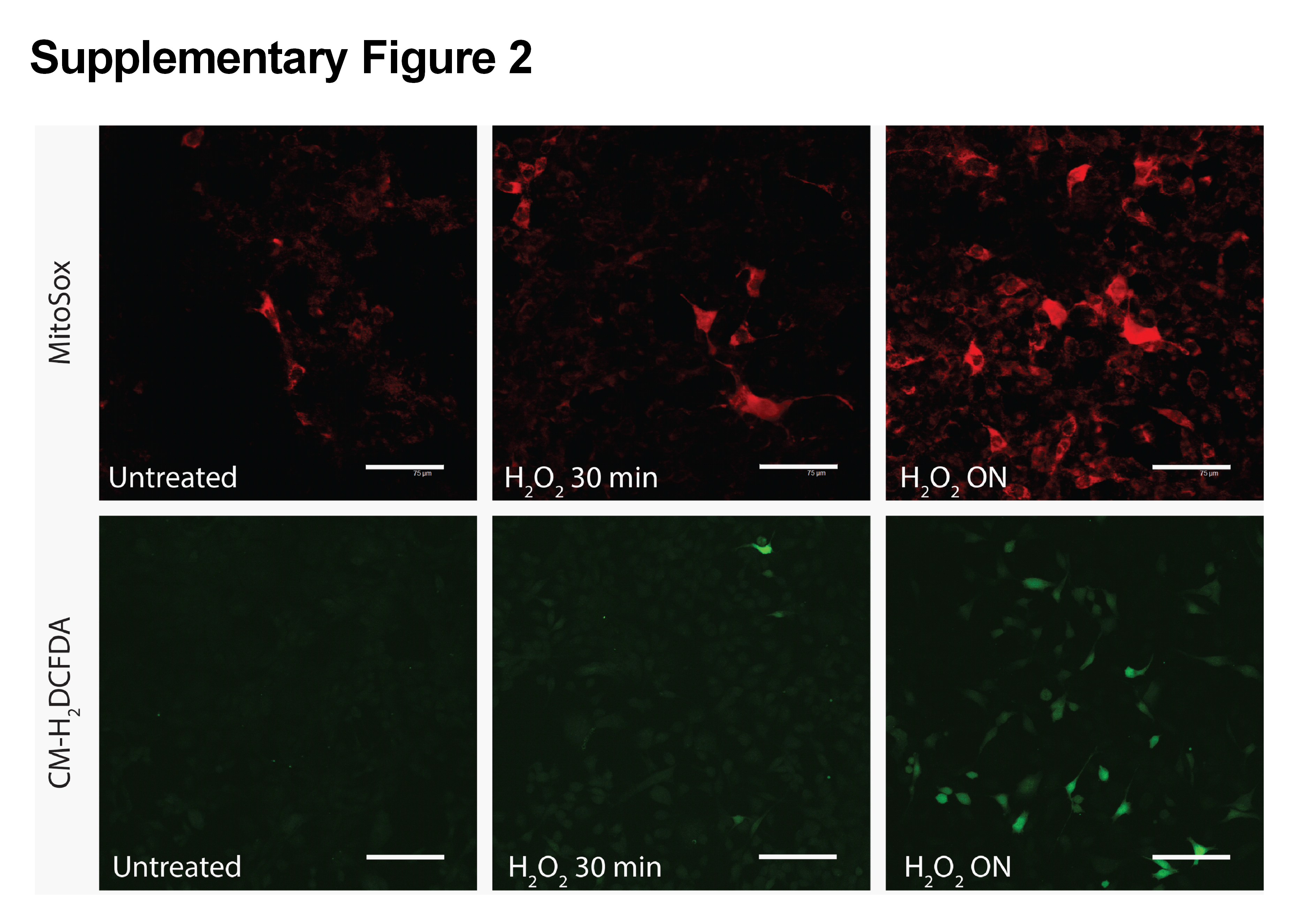

Supplement: Supplementary file 6 — Figure S2. Oxidative stress in CT26 cells. Representative staining of CT26 cells with the fluorogenic dyes MitoSOX and CM-H2DCFDA after 30 min and O/N treatment with 200 μM H2O2. Magnification: 40X. (TIF 7723 kb) [file 13046_2019_1205_MOESM6_ESM.tif]

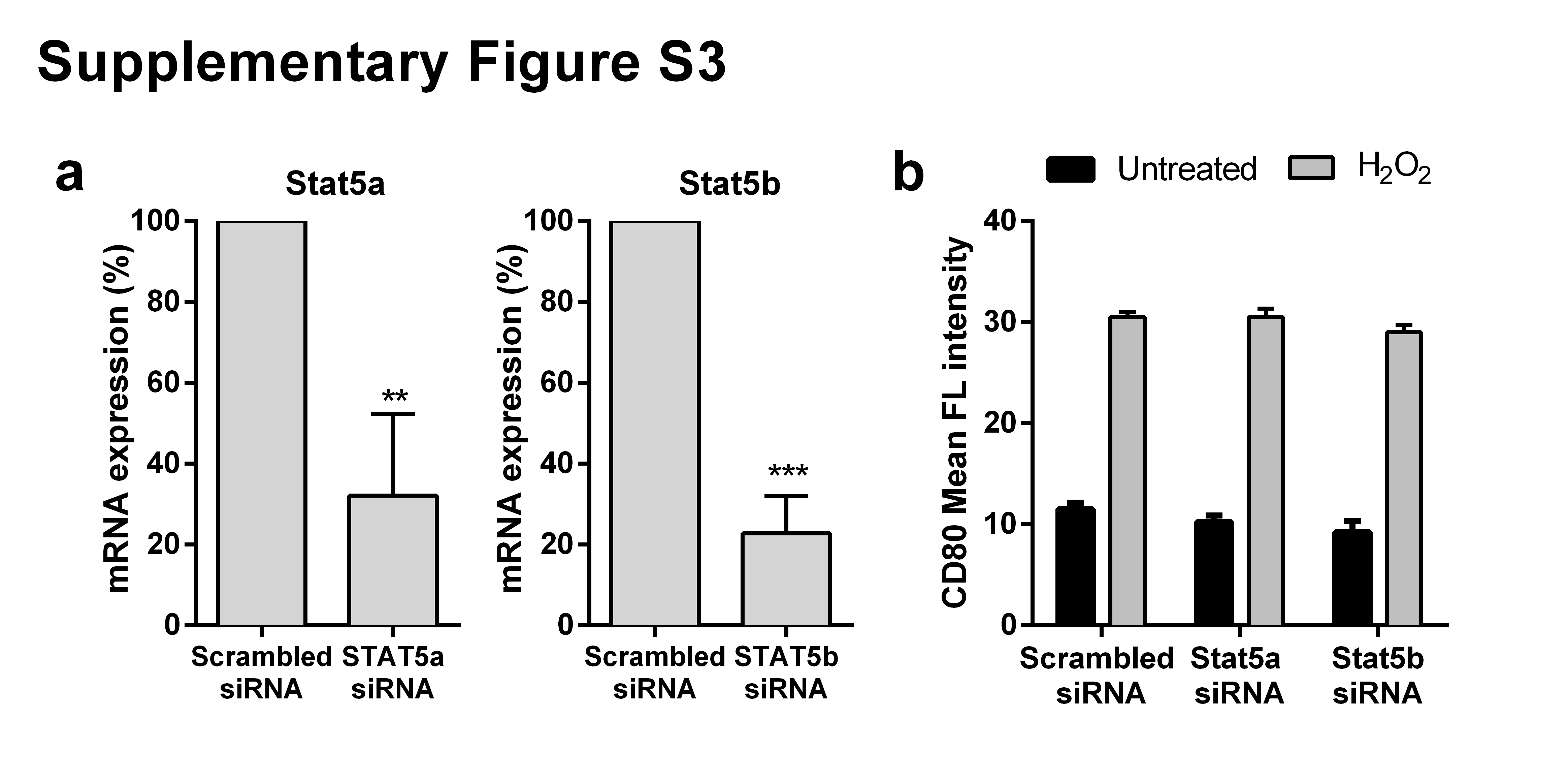

Supplement: Supplementary file 7 — Figure S3. CD80 induction by oxidative stress is not mediated by STAT5. (a) CT26 cells were transfected with control, STAT5a or STAT5b siRNAs. After 24 h, silencing efficiency was tested by RT Real Time PCR. (b) CT26 cells were transfected with control, STAT5a or STAT5b siRNAs. After 24 h, cells were treated with 200 μM H2O2 for 24 h before flow cytometry for CD80. Data are presented as mean ± S.E.M. **P < 0.01 *** P < 0.001 by unpaired, two-tailed Student’s t-test. (TIF 280 kb) [file 13046_2019_1205_MOESM7_ESM.tif]
